# Compliance with Surgical Safety Checklist completion in the operating room of University of Gondar Hospital, Northwest Ethiopia

**DOI:** 10.1186/s13104-015-1338-y

**Published:** 2015-08-19

**Authors:** Tadesse B. Melekie, Gashaw M. Getahun

**Affiliations:** Department of Medical Anesthesiology, College of Medicine and Health Sciences, University of Gondar, Gondar, Ethiopia; Department of Surgery, College of Medicine and Health Sciences, University of Gondar, Gondar, Ethiopia

**Keywords:** Implementation, Surgery, Safety, Sign-in, Time-out, Sign-out

## Abstract

**Background:**

Appropriate utilization and compliance of Surgical Safety Checklist reduces occurrence of perioperative surgical complications and improve patient outcomes. However, data on compliance of surgical checklists are scarce in the study area. Therefore, the aim of this study was to evaluate compliance of checklist completion and its barrier for utilization at University of Gondar Hospital, Northwest Ethiopia.

**Methods:**

A prospective observational study was conducted among 282 patients undergoing elective and emergency surgery from January to March 2013. Compliance and completeness rate with implementation of Sign-in, Time-out, and Sign-out domains was computed with SPSS 20 package.

**Results:**

A total of 282 operations were performed and checklists were utilized in 39.7 % (112/282) of cases. Among these, most checklists were employed during emergency procedures (61.6 %) that need general anesthesia (75.9 %) in department of surgery (58.9 %). The overall compliance and completeness rate were 39.7 and 63.4 % respectively. The sign-in, time-out and sign-out were missed in 30.5 % (273/896), 35.4 % (436/1,232) and 45.7 % (307/672) respectively. The main reasons cited for non-user were lack of previous training (45.1 %) and lack of cooperation among surgical team members (21.6 %).

**Conclusions and recommendations:**

The completeness rate was satisfactory but the overall compliance rate was suboptimal. An instrument that is used 40 % of the time has been a fairly basic introduction without significant reinforcement training. Moreover, frequent use of the checklist during emergency cases has been deemed to be of value by clinicians. Supplementary training and attention to actual checklist use would be indicated to ensure that this valuable tool could be used more routinely and improve communication. Conducting regular audit of checklist utilization is also recommended.

## Background

Surgical service is one of the fundamental health care services given in the healthcare system [1]. Over 234 million surgical operations are performed annually worldwide and complications are occurred in 3–16 % of surgical procedures [1, 2]. Surgical complications are a major cause of morbidity and mortality and also pose a major financial burden to patients and providers [3]. But it has been estimated that at least half of the complications that occur are avoidable [3, 4]. The importance of a strong safety culture that enhances patient safety initiatives has been reiterated for years in the healthcare system and the safety of surgical care therefore is a global concern [5].

Evidence showed implementation of different effort modalities for an improved surgical outcome [6–8]. As part of its efforts to improve patient safety, the World Health Organization launched Safe Surgery Saves Lives programme in 2008. The aim was to harness political commitment and clinical will to address important patient safety issues, including inadequate anesthetic safety practice, avoidable surgical infection and poor communication among team members [3, 9, 10]. Since the development of checklists for use in operating rooms, their use (compliance to all the three time frame) has become greater than ever and associated with a significant reduction in postoperative complications and mortality [11–13]. Recently, however, questions have arisen about their ease of introduction into workflow patterns and their true impact on safety [14].

To establish highly successful implementation processes of Surgical Safety Checklist, every health care provider including hospital managers, have to actively lead the processes [15, 16], deliberately enroll to the work [17, 18], create an extensive multidisciplinary discussion and communication, arrange update trainings [11, 19], offer ongoing constructive feedbacks and conduct regular audits [20–22]. In general, implementing Surgical Safety Checklists needs high level interaction among social, cultural, and operational reasons in the health system [23, 24].

Ethiopia, one of the developing countries with a population of 82.8 million [25], currently undergoing extensive development of healthcare services. Data on surgical outcomes is limited but published figures showed an all-cause surgical mortality of 7 % [26]. The Clinton Health Access Initiative (CHAI) and Yale Global Health Leadership Institute (GHLI) are working together with the Ethiopian Federal Ministry of Health to improve healthcare services across Ethiopia through the introduction of Ethiopian Hospital Reform Implementation Guidelines (EHRIG) in 2010 [27]. The WHO Surgical Safety Checklist is an important tool and its introduction to Ethiopian hospitals is an integral part of the EHRIG. While critics point out that checklists alone are not sufficient to improve patient safety, and must be accompanied by wider strategies for quality improvement, it is hoped that implementation of the checklist will reduce surgical mortality and morbidity [28, 29]. The benefits of the Checklist, however, depend upon the individual hospitals’ ability to implement it effectively.

In January 2010, African Partnership for Patient Safety (APPS) project introduced the implementation of a locally modified surgical checklist, an inexpensive and easy-to-use tool, in University of Gondar Hospital. Prior to implementation, surgical teams (surgeons, gynecologist/obstetricians, residents, anesthetists and nurses) and hospital administrators have been trained on general principle of patient safety and implementation issues. Since then however, its consistent use, compliance and barriers for utilization was not evaluated. Therefore, the main objectives of this study were to evaluate checklist compliance and identify challenges and barriers in utilization of checklist at University of Gondar Hospital, through direct observation of the perioperative checklist process.

## Methods

A prospective observational study was conducted at University of Gondar Hospital from January to March 2013. It is a 500-bed tertiary university hospital serving a population of 5 million inhabitants. In 2010, an interdisciplinary team consisting of anesthetists, surgeons, resident physicians and operating theater nurses adapted the WHO checklist to local circumstances. The circulating nurse was designated as the checklist coordinator to guide the team throughout all questions and ticked the checkboxes. But the sign-in, time-out and sign-out phase had to be initiated/checked by anesthetists and residents or/and operating nurses respectively. The checklist coordinator was obliged to only tick the checkbox if an answer was given to the corresponding question. Finally, the checklist becomes part of the patient’s paper-based notes and attached with medical record of patient. Prior to the first attempt to use the checklist in the operation room, the corresponding operating teams were given 2 days introduction training with practical demonstration. Since then, formal update training was not given for surgical teams. New staffs had been introduced to the checklist by respective heads before joining the surgical team. Hospital administrators were also included in the project and have travelled for benchmarking to high and low- income countries.

After 4 month piloting, all checklist were collected and checklist use for each operation was noted. It was as whether checkboxes were ticked as required. Results were discussed with the operating team, department leaders and hospital managers. Starting in June 2010, operating staffs were prepared for checklist introduction in operation room. Thereafter, the team decides to utilize the checklist which contains a total of 25 items covering three timeframes: before anaesthesia (‘sign in’—eight items), before skin incision (‘time out’—eleven items), and before leaving the operating room (‘sign out’—six items).

The primary outcome measures were whether the checklist was generally used and the respective completion rate in case of using a checklist. Data on compliance and completeness were collected by clinical nurses who are working in the hospital after a 1-day training session. Date of operation, procedure, type of surgery (emergency or elective), shift (day or night), type of anesthesia, and adherence to the Surgical Safety Checklist (sign-in, time-out, and sign-out) were collected from all discharged surgical patient. A complete checklist is defined as a checklist in which all items have been ticked. Checklists were kept as part of each patient’s medical record. However, as medical records were not completely computerized, checklist data could not be extracted automatically. All consecutive postoperative patients (N = 282) were included during study period and analyzed. Secondary outcome measures were information on the barriers and challenges arising during its implementation/utilization process and the frequency of use as self-reported by the respondent. It was collected from all members (N = 82) of the hospital surgical team i.e. physicians (n = 35), anesthetists (n = 20) and operation room nurses (n = 27) using structured self administered questionnaires.

Data were coded, cleaned, entered and analyzed using SPSS Version 20. Descriptive statistic was used to display checklist compliance and completeness. Categorical variables are presented as absolute and relative frequencies; for metric variables median and range (minimum, maximum) are given as none of these variables was normally distributed.

Ethical clearance and official permission was secured to conduct the study from ethical board of University of Gondar and medical director of University of Gondar Hospital respectively.

## Results

### Compliance of use and checklist completeness

During the study period, 282 operations were performed with spinal and general anesthesia. Checklist was used in 39.7 % (112/282) of operations; within used checklists, 63.4 % (71/112) were complete (i.e. all items of the checklist had been ‘ticked off’) and 36.6 % (41/112) were partially complete (i.e. all items of the checklist have not been ‘ticked off’). As a result, the overall compliance and completeness rate were 39.7 and 63.4 % respectively.

As shown in Table 1, most checklists were employed in department of surgery (58.9 %), during the day shift (53.6 %), in patient who undergone emergency surgery (61.6 %) and in procedures involving general anesthesia (75.9 %).

**Table 1:** Surgical Safety Checklist utilization among operated surgical patients at University of Gondar hospital, 2013(N = 282)

Variables	Use of Surgical Safety Checklist		
	Yes^b^ (n = 112)	No (n = 170)	Total operated (n = 282)
Age			
5–20	24 (21.1)	28 (16.5)	52 (18.4)
21–40	38 (34.0)	78 (45.9)	116 (41.2)
≥41	50 (44.9)	64 (37.6)	114 (40.4)
Sex			
Male	66 (58.9)	85 (50.0)	151 (53.5)
Female	46 (41.1)	85 (50.0)	131 (46.5)
Type of anesthesia			
General	85 (75.9)	112 (65.9)	197 (69.9)
Spinal	27 (24.1)	58 (34.1)	85 (30.1)
State of surgery			
Elective	43 (38.4)	66 (38.8)	109 (38.7)
Emergency	69 (61.6)	104 (61.2)	173 (61.3)
Time of operation			
Day	60 (53.6)	71 (41.8)	131 (46.5)
Night	52 (46.4)	99 (58.2)	151 (53.5)
Type of procedure			
Obstetrics and gynecology	46 (41.1)	37 (21.8)	83 (29.4)
Surgery^a^	66 (58.9)	133 (78.2)	199 (70.6)

^a^Surgery—urology, head, neck and breast, vascular, plastic surgery, abdominal, orthopedic, ear–nose–throat surgery

^b^Does not mean that all items on all three parts of the checklist have been ‘ticked off’

### Analysis of phases and individual items of the checklists

The analysis included 112 surgical procedures. Overall, 112 checklists were handed in and 2800 items were analyzed to find out which items were most commonly used/missed. From these check items evaluated, 36.3 % (1016/2800) were missed. The most frequently missed checklist items were item 23 (99 times), 17 (71 times) and 5 (69 times) that state “surgeon, anesthesia professional and nurse review the key concerns for recovery and management of this patient”, nurse verbally confirms with the team whether there are any equipment problems to be addressed” and whether the patient have a known allergy or not respectively (Table 2). The sign-in, time-out and sign-out were missed in 30.5 % (273/896), 35.4 % (436/1232) and 45.7 % (307/672) respectively.

**Table 2:** Missing items in Checklists at University of Gondar Hospital, January–March, 2013 Northwest Ethiopia

Item no.	Checklist items	Number of times missing	%
Sign in			
1	Has the patient confirmed his/her identity, site, procedure and consent?	19	1.9
2	Is the site marked?	61	6.0
3	Are the anesthesia equipment and medication checks complete?	6	0.6
4	Pulse oximetry is attached and functional	6	0.6
5	Does the patient have a known allergy?	69	6.8
6	Does the patient have a difficult airway or aspiration risk?	34	3.3
7	Is risk of blood loss >500 ml and require blood?	55	5.4
8	Does the assigned person put his/her name and signature?	23	2.3
Subtotal		273	26.9
Time out			
9	Confirm all tem members have introduced themselves by name and role	63	6.2
10	Confirm the patient’s name, procedure and site of incision	16	1.6
11	Has antibiotic prophylaxis been given within the last 60 min?	28	2.8
Anticipated critical events to surgeon:			
12	What are the critical or non-routine steps	23	2.7
13	How long will the operation take?	42	4.1
14	Is the anticipated blood loss > 500 ls?	33	3.2
Anticipated critical events to anesthetist:			
15	Are there any patient-specific concerns?	61	6.0
Anticipated critical events to nursing team:			
16	Sterility confirmed	2	0.2
17	Are there equipment issue or any concern	71	6.7
18	Is essential imaging displayed	53	5.2
19	Does the assigned person put his/her name and signature?	44	4.3
Subtotal		436	43.0
Sign out			
20	Nurse verbally confirms name of procedure	57	5.6
21	Completion of instrument, sponge, needle and suture counts	3	0.3
22	Are there any equipment problems to be addressed?	52	5.1
23	What are the key concerns for recovery and management of this patient?	99	9.7
24	Specimen labeled correctly	41	4.0
25	Does the assigned person put his/her Name and signature?	55	5.4
Subtotal		307	30.1
Total		1016	100

### Before induction (sign-in period)

In this period, 83.0 % of the patients were confirmed on his/her identity, site, procedure and consent. Anesthetic machines, equipments and drugs were checked in 94.6 % of the cases and corrective measures were taken for 3.4 % of cases. Oxygen saturation measurement instrument, pulse oximetry, was attached to the patient and was functional in 94.6 % cases. Every case was assessed for potential drug allergy (38.4 %); difficult airway, risk of aspiration (69.6 %) and anticipated blood loss. In line with these, appropriate protective measures were taken for every identified risk which was reminded by the checklist (Table 2).

### Before skin incision (time-out period)

Surgical teams were introduced themselves by name and role in only 43.8 % of the cases. But the patient’s identity, operative site, and type of procedure performed were confirmed in 85.7 % of the cases. Twenty-five percent of the cases were identified by the checklist and antibiotic prophylaxis was administered 1 h before incision (Table 2).

### Before patient left operating room (sign-out period)

In sign-out period, the result depicted that nurses verbally confirmed the names of performed procedure in 49.1 % of the cases. But materials used for the operations were counted before the closure of the incision in 97.3 % of the cases. On the other hand, the surgical teams discussed the main concerns of recovery room condition and patient management in 11.6 % of cases (Table 2).

### Reasons for non-utilization of checklist

Surgical teams working in the hospitals were asked regarding whether they utilize/assist to utilize the checklist or not since 2010. They also asked about the barriers which affect the utilization and implementation process. All the self administered questionnaires were returned making the response rate of 100 %. As depicted in Table 3, among the respondents (N = 82), only 31 (37.8 %) were involved in the implementation process (either utilize themselves or assist others) and majority (62.2 %) did not use the Surgical Safety Checklist or parts of it at all. Of the non-user, majority were nurses (41.2 %), residents (39.2 %) with a service of less than or equal to two (56.9 %) years. The main reasons cited by non-user participants were lack of previous training (45.1 %) followed by uncooperative surgical teams while implementation or using the checklist (21.6 %).

**Table 3:** Characteristics and barriers in utilization of Surgical Safety Checklist in University of Gondar hospital, 2013(N = 82)

Variables	Ever utilize/assist		
	No (n = 51)	Yes (n = 31)	Total
Profession			
Nurses (n = 27)	21 (41.2 %)	6 (19.4 %)	27
Anesthetists (n = 20)	11 (21.6 %)	9 (29.0 %)	20
Physicians (n = 35)	19 (37.3 %)	16 (51.6 %)	35
Level of education			
BSc	28 (54.9 %)	15 (48.4 %)	43
MSc	2 (3.9 %)	2 (6.4 %)	4
GP	5 (9.8 %)	2 (6.4 %)	7
Residents	10 (19.7 %)	8 (25.8 %)	18
Seniors	4 (7.8 %)	6 (19.4 %)	10
Year of services			
≤2 year	29 (56.9 %)	9 (29.0 %)	38
2–5 years	13 (25.5 %)	12 (38.8 %)	25
≥5 years	9 (17.6 %)	10 (32.2 %)	19
Reasons for non implementation			
Unavailable of checklists	7 (13.7 %)		
Have no formal training before	23 (45.1 %)		
Time constraint	10 (19.6 %)		
Uncooperative surgical teams	11 (21.6 %)		

## Discussion

The implementation of a checklist is intended to improve the outcome of surgical care and thus the quality of care in general. However, its introduction and sustainability is always a big challenge. Of course, the translation of a new concept into practice usually follows theory of diffusion and innovation—acquire knowledge, persuaded by utility, make a decision to adopt, determine usefulness, and then decide to continue using the innovation to full effect [30]. In this observational study we have found, although the hospital reported 100 % utilization of the checklist in the operation room, a 40 % compliance with a varying degree of completeness of items (63.4 %). This is quite good that an instrument that is used 40 % of the time 3 years after what appears to have been a fairly basic introduction without significant reinforcement training.

Sign-in period was relatively administered in a higher rate (69.5 %), of which the greatest fulfillment was anesthetic equipment and medication checking. These items are important in preventing the most common errors that causes serious harm to the patient [31]. Moreover, functional pulse oximetry was attached in the majority of the cases which helps to detect desaturation at the early stage. In contrast, items of aspiration risk, anticipation of a difficult airway, allergic history and estimated blood loss were found unchecked in most cases, all of them could lead to loss of life [32].

Surgical team communication is one of the key intentions of the WHO Surgical Safety Checklist [33]. In Time-out period, surgical teams are expected to introduce each other by name and functional role. Nevertheless, the findings of this study showed that only 56.3 % of team members were introduced themselves by names and roles. The result is similar with study conducted in Thailand in which majority of the surgical team failed to introduce their name and functional role to others [34]. The reason might be explained by surgical teams were communicated and introduced themselves for a long period of time in their practical place. Moreover, people often introduce each other only during the first contact. In this respect, many studies depicted that serious complications could occur when there are unsuccessful communication and cooperation among the surgical team members [35].

In this study finding, Sign-out period was poorly performed (54.3 %) compared with other sections. This is consistent with experience from the UK and Thailand hospitals [14, 34]. The potential causes for this period could be tightly preoccupied surgical teams (nursing teams with final instrument count, processing and preparation for the next case, surgical and anesthetic teams with patient extubation, oxygen preparation in recovery room, procedure note writing and patient transfer) during that procedure.

Communication errors are the most common cause of adverse events in healthcare. For instance, information does not reach the right person, or is inaccurate, or issues remain unresolved until they become critical. In the operating theatre, this leads to mistakes, inefficient use of resources, wasted equipment, frustration, poor morale and delays [36]. This problem was in line with current study finding, as the main reason cited was uncooperativeness of surgical team while filling the checklist and lack of previous training, both of them are sources of communication error. Literature indicates that over time, compliance of surgical staff is good but needs follow up and sustained education sessions including meetings to review and address the barriers in a comprehensive way [9, 22].

The importance of local champions was highlighted and effective implementation was seen when senior clinicians showed good leadership skills, demonstrated how to use the checklist, and explained why it was necessary [37]. But this study showed that more than half (19/35) of the physicians were not consistently utilized the checklist. This may have impact on patient outcome and not being exemplary for other staffs. It appears that it is not only the technical skill, but also the behavioral patterns and non-technical skills of the physician/surgeon (leadership, teamwork, problem-solving, decision-making & situation awareness), that affect surgical outcomes [37, 38].

## Limitation of the study

This study has some limitations. It was conducted in only one setting and in a brief period of time which comprise of relatively small sample; therefore, the results might not be applicable to other settings throughout the country. Moreover, the study relies on data from the patients’ medical records and validation of checklist utilization is not presented. The authors did not make direct observations during the procedure which may cause Hawthorne effect.

## Conclusion and recommendations

Despite checklist was not used in all operations, all the three parts (all items) of the checklist had been ‘ticked off’ in majority of the operations among those who utilized the checklist. As a result, the completeness rate was satisfactory but the overall compliance rate was suboptimal. The present study did not assess outcomes, but it is assumed that poor compliance puts patients at risk. The checklists were used more frequently during daytime in emergency patients who took general anesthesia. Sign-in and Time-out period were performed in satisfactory manner yet it was not performed with equal frequency in all aspects of the items. The Sign-out section was clearly seen as more difficult, and less important, to complete than other sections. The main reasons cited while utilizing the checklist were lack of previous training and uncooperative surgical teams. Regular and appropriate implementation of checklist is used as a tool for improving team communication; strengthening teamwork and improving patient safety. On top that, to amplify consistency, the active team members should be motivated to utilize the checklist during their work practice regularly. Awareness creation should be in place especially for new nursing/anesthetic staffs because of high turnover. Moreover, conducting regular audit of checklist utilization, offering regular refreshment and multidisciplinary training to improve communication may increase the rates of compliance with the checklist. Supplementary training and attention to actual checklist use would be indicated to ensure that this valuable tool could be used more routinely.
